# Interprofessional collaborative practice within cancer teams: Translating evidence into action. A mixed methods study protocol

**DOI:** 10.1186/1748-5908-5-53

**Published:** 2010-07-13

**Authors:** Dominique Tremblay, Danielle Drouin, Ariella Lang, Danièle Roberge, Judith Ritchie, Anne Plante

**Affiliations:** 1 Centre de Recherche Hôpital Charles LeMoyne, Greenfield Park, Quebec, Canada; 2 Faculty of Medicine and Health Sciences, Université de Sherbrooke, Longueuil, Québec, Canada; 3 Hôpital Charles LeMoyne, Centre intégré de cancérologie de la Montérégie, Greenfield Park, Quebec, Canada; 4 Victorian Order of Nurses, VON Canada National Office, Ottawa, Ontario, Canada; 5 McGill University Health Centre & School of Nursing, McGill University, Montreal, Quebec, Canada

## Abstract

**Background:**

A regional integrated cancer network has implemented a program (educational workshops, reflective and mentoring activities) designed to support the uptake of evidence-informed interprofessional collaborative practices (referred to in this text as EIPCP) within cancer teams. This research project, which relates to the Registered Nurses' Association of Ontario (RNAO) Best Practice Guidelines and other sources of research evidence, represents a unique opportunity to learn more about the factors and processes involved in the translation of evidence-based recommendations into professional practices. The planned study seeks to address context-specific challenges and the concerns of nurses and other stakeholders regarding the uptake of evidence-based recommendations to effectively promote and support interprofessional collaborative practices.

**Aim:**

This study aims to examine the uptake of evidence-based recommendations from best practice guidelines intended to enhance interprofessional collaborative practices within cancer teams.

**Design:**

The planned study constitutes a practical trial, defined as a trial designed to provide comprehensive information that is grounded in real-world healthcare dynamics. An exploratory mixed methods study design will be used. It will involve collecting quantitative data to assess professionals' knowledge and attitudes, as well as practice environment factors associated with effective uptake of evidence-based recommendations. Semi-structured interviews will be conducted concurrently with care providers to gather qualitative data for describing the processes involved in the translation of evidence into action from both the users' (n = 12) and providers' (n = 24) perspectives. The Graham *et al. *Ottawa Model of Research Use will serve to construct operational definitions of concepts, and to establish the initial coding labels to be used in the thematic analysis of the qualitative data. Quantitative and qualitative results will be merged during interpretation to provide complementary perspectives of interrelated contextual factors that enhance the uptake of EIPCP and changes in professional practices.

**Discussion:**

The information obtained from the study will produce new knowledge on the interventions and sources of support most conducive to the uptake of evidence and building of capacity to sustain new interprofessional collaborative practice patterns. It will provide new information on strategies for overcoming barriers to evidence-informed interventions. The findings will also pinpoint critical determinants of 'what works and why' taking into account the interplay between evidence, operational, relational micro-processes of care, uniqueness of patients' needs and preferences, and the local context.

## Background

### Context

Most cancer and palliative/end-of-life programs propose interprofessional collaboration as a key modality for improving quality of care [1-5]. The need for greater collaboration is being driven by the same pressures as those driving the cancer services transformation agenda: the pressure for timely access to care, lack of continuity in care, needs unmet by current services, demand for supportive care and dearth of health human resources. To grapple with these issues, the regional cancer network in Montérégie, a region in Quebec, Canada, has implemented a program designed to expand existing interprofessional collaboration among nurses, doctors, and other care providers (pharmacists, nutritionists and social workers) working on cancer teams.

The development of the 'Psychosocial oncology: Building interprofessional capacity to improve cancer care across the continuum' program (referred to here as the POBC^3^) was a nurse-led interdisciplinary project that was funded by the Canadian Partnership against Cancer. A summary of the program components is presented in Table 1. This program, which is related to recommendations made in the Registered Nurses' Association of Ontario (RNAO) Best Practice Guidelines [6,7] and other sources of evidence [8-10], represents a unique knowledge transfer initiative enabling local cancer team members to experiment with EIPCP. We will use this program to systematically examine the factors and processes involved in the adoption of evidence-based recommendations and their adaptation into practices.

**Table 1:** POBC multiple strategies and recommendations from RNAO documents

**Improvement initiative POBC**^**3 **^**multiple strategies**	RNAO/BPG Recommendations
Participants discuss the components of collaborative practice to understand what is involved and the underlying arguments. This intervention arouses professionals' interests and helps to determine goodness of fit with their local work environment.	Develop knowledge about the values and behaviours that support teamwork and the impact of teamwork on patient/client safety and patient/client outcomes. As such, nurses:
	▪ Inform themselves about the attributes of supportive teams.
	▪ Articulate their belief in the value of teamwork.
	▪ Demonstrate their willingness to work effectively with others.

Participants are involved in reflective communication exercises, and diverse educational strategies are employed to develop their relational capacities. This strategy identifies enablers of and barriers to effective communication.	Contribute to a culture that supports effective teamwork by:
	▪ Demonstrating accountability for actions, enthusiasm, motivation, and commitment to the team.
	▪ Understanding own roles, scope of practice, and responsibilities, as well as seeking information and developing an understanding about other roles and scopes of practice.
	▪ Being accountable for and respectful in the manner in which they communicate.

Once participants identify a clinical situation of interest, they discuss psycho-social interventions in a collaborative way. Activities are conducted to ensure assimilation of the core concepts by the participants in collaboration with a psychosocial expert and two regional, trained professionals.	Teams establish clear processes and structures that promote collaboration and teamwork that leads to quality work environments and quality outcomes for patients/clients by:
	▪ Establishing processes for conflict resolution and problem solving.
	▪ Establishing processes to develop, achieve, and evaluate team performance, common goals, and outcomes.
	▪ Building capacity for systematic problem solving.
	▪ Participating to the implementation of practices to support enhanced collaboration at the functional and organizational level.
	▪ Incorporating non-hierarchal, democratic working practices to validate all contributions from team members.
Mentoring by professional experts target problem-solving strategies, conflict resolution strategies to ensure sustainability of learning in doing, and identify needs for further educational workshop.	▪ Incorporating processes that support continuity of care with patients/clients to enhance staff satisfaction, staff self-worth, and patient/client satisfaction.
	▪ Establishing processes for decision making for a variety of circumstances such as:
	• emergencies;
	• day-to-day functioning;
	• long-term planning;
	• policy development;
	• care planning

Assess participants' perceptions of their current inter-professional functioning and provide feedback to each other.	Teams establish processes which promote open, honest, and transparent channels of communication by:
	▪ Establishing processes to ensure effective communication.
	▪ Developing skills in active listening.

We define EIPCP as a transformative model for cancer services delivery that engages care providers in the 'process of working together to build consensus on common goals, approaches and outcomes. It requires an understanding of own [sic] and others' roles, mutual respect among participants, commitment to common goals, shared decision making, effective communication, relationships and accountability for both the goals and team members' [6]. EIPCP entails proactive strategies that make care providers aware of evidence-based recommendations and facilitate the translation of this knowledge into day-to-day practice, as a basis for quality-of-care improvement.

### Translating evidence into action

The gap between research evidence on interprofessional collaboration and practice is wide, well documented [8,11], and troubling, especially in cancer services where the cancer crisis jeopardizes the ability of health systems to respond to patients' needs [1]. Even though collaboration benefits users, providers, and organizations [12-14], many professionals only pay lip service to the premise of collaborative practice [15-17]. Previous studies have emphasized key enablers of and barriers to interprofessional collaboration: a lack of consensus about terminology, the need for interprofessional collaboration initiatives to have champions and external support, sensitivity to the effects of profession-related cultures, and the logistics of implementation [18]. Other barriers include structural issues such as competition between professionals, and conceptual problems such as a lack of understanding of mutual roles and a lack of experience or training in interdisciplinary collaboration among providers [14]. Less is known about how evidence-based recommendations could be adopted and adapted by care providers to overcome those barriers. Moreover, the preferences of those using such services with regard to the ways professionals work together and share the clinical information are poorly understood and are understudied.

### Theoretical background to use of research evidence

**Figure 1:**
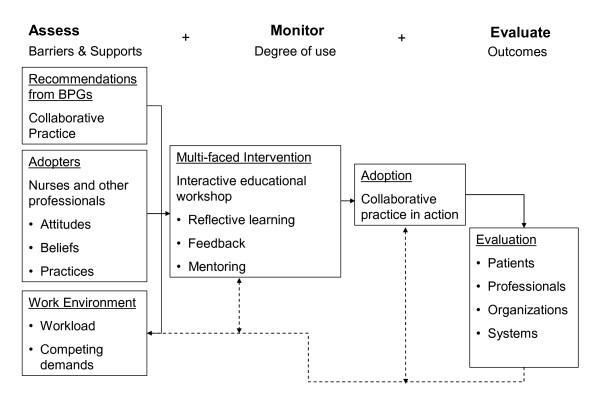
**Theoretical framework**. Adapted from Graham & Logan [19].

Multiple, interacting conditions pose a challenge to the utilization of research findings. For practical reasons, these conditions could be grouped under six main elements as proposed in the comprehensive Ottawa Model of Research Use (OMRU) [19]. This model is an interdisciplinary framework presenting the utilization of research evidence as a dynamic process based on multiple, interrelated decisions and actions. It has provided guidance for numerous studies [20-22]. The OMRU points to the importance of assessing barriers to the translation of knowledge into action at three levels: the characteristics of the recommendations made in the guidelines (*e.g*., perceive usefulness, fit with current practice, norms/values), the characteristics of the professionals involved (*e.g*., awareness, attitudes, knowledge/skills, concerns, current practice) and the characteristics of the practice environment (*e.g*., users' preferences, work pressure, competing demands/time) (Figure 1).

The literature on innovation and change in health services [23-29] and RNAO documentation [6,30] will also serve as conceptual background for our study. Our literature review led us to factor in the following four points: the novelty of EIPCP should be considered from the perspectives of the individuals who experiment with new ways of doing things; the players in the field have their own interpretations of evidence and their own definitions of the weaknesses in their practices--they therefore have intuitive ideas of what should be done to improve care delivery, but it is difficult for them to drive change within the context of their day-to-day activities; the mobilization of multiple actors with different areas of expertise and resources around a specific project focuses their efforts and provides a better chance of success; and the evaluation of an innovation contributes to its success--it allows the professionals involved in reflective activities to develop their receptive capacity through doing. Overall, the literature on innovation was used to define more clearly the research aim and specific objectives. It will also support data analysis, which will consist of matching empirically observed EIPCP translation events to the theoretically predicted elements identified earlier.

### Research aim and objectives

This study aims to examine the uptake of evidence-based recommendations from best practice guidelines intended to enhance interprofessional collaborative practices within cancer teams. In this study, care providers are those directly or indirectly (clinicians, managers, decision-makers responsible for governance) involved along the cancer trajectory, including community/home care, specialized hospital and ambulatory cancer services, as well as palliative/end-of-life care.

More specifically, our study objectives are as follows: to assess how professional knowledge, beliefs and the practice environment support or impede the adoption of EIPCP; to assess how patients' knowledge, beliefs and needs influence this adoption process; and to describe the impact of an educational workshop and mentoring program on the uptake and sustainability of EIPCP over a six- to eight-month period.

## Methods

### Design

**Figure 2:**
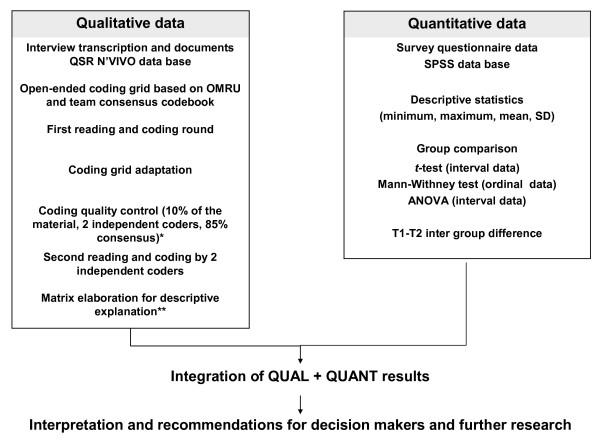
**Mixed methods integration**. * Saldana [47]. ** Miles & Huberman [46].

The planned study constitutes a practical trial, defined as a trial designed to provide comprehensive information that is grounded in real-world healthcare dynamics [31]. An exploratory mixed methods design will be used [32]. This design will involve concurrent quantitative and qualitative data collection and analysis. The quantitative and qualitative results will then be merged to provide complementary perspectives. The mixed methods design will in turn allow us to form a more complete picture of the interrelated contextual elements and the associated individual characteristics that determine EIPCP than use of a single method would allow (Figure 2) [33].

### Participants and recruitment

The participants will be selected from two groups. The care provider group (PG) will consist of professionals, managers, and decision-makers responsible for governance at the regional level who are directly or indirectly involved in the POBC^3 ^improvement initiative. The user group (UG) will consist of patients/caregivers using services at one of four care settings in the Montérégie cancer network that are involved in the POBC^3^. Inclusion criteria for the PG will include being: a key stakeholder knowledgeable about POBC^3 ^planning, implementation and/or evaluation, and a participant in the workshop and/or mentoring activities of the POBC^3^. Inclusion criteria for the UG will include being 18 years of age or older, able to understand and communicate in French, and a current user of cancer services at one of the participating care settings. The potential PG participants will be recruited during the POBC^3 ^workshop. They will be asked if they agree to be contacted for a study on EIPCP and to provide their contact information. For the UG group, field professionals will be asked to identify potential participants for interviews using a purposive sampling strategy aimed at maximum variability (*e.g*., age, gender, tumour site, stage on the cancer trajectory) [34]. For users interested in participating, a nurse who has extensive experience with cancer patients will make the initial contact to obtain their informed consent and conduct the interview.

### Data collection

To meet objective one, quantitative data will be collected from professionals using an adapted cancer care version of the survey questionnaire from Davies' study on collaborative maternity care [35]. This questionnaire was constructed on the basis of the literature, which suggested that the following issues should be considered in developing a survey tool in this field: the concepts that should be demonstrated in a collaborative practice and the importance of the components of a collaborative practice model. The questionnaire also includes the Attitude toward Health Care Teams Scale [36], which contains two subscales: quality of care/process (14 items, Cronbach's alpha = 0.83) and physician centrality (six items, Cronbach's alpha = 0.68). Finally, a subscale (five items) taken from the Interprofessional Collaboration Questionnaire [37] will be used to assess the intensity of collaborative practices. These questionnaires provide operational measures that will be used to assess potential adopters' knowledge, attitudes, beliefs and current practice as described in the OMRU. To meet objective three, which focuses on the sustainability of practice over time, the questionnaire will be administered at the end of the POBC^3 ^two-day workshop (T1) and six to eight months later after a period of mentoring (T2). This time frame was adopted first because the POBC^3 ^is currently underway and pre/post-measurements are therefore not possible. Second, it was important to ensure a minimal sample size at T2 by taking potential workforce turnover into account.

Concurrently with T2, we will collect qualitative data to deepen our understanding of service users' perspectives (objective two), the contextual factors and the processes determining EIPCP patterns. In-depth interviews (60 minutes) will be conducted to gain understanding of the experience, the challenges, and the insights of both service users (n = 16) and care providers (n = 24). We will use a systematic interview guide adapted from Edwards' [38] and Peterson's [17] previous works on best practice guidelines implementation. The interviews will be audio-taped. With assistance from the care settings, we will purposefully select archival material that provides records of the EIPCP process. By way of example, documents will include reports, protocols and procedures, aggregated and non-identifiable patient reports, information sheets, minutes of meetings, and other relevant print material. No information from individual patient records will be collected.

### Sample size

Approximately 100 professionals and managers are involved in POBC^3 ^activities. Based on our previous studies [39,40], we anticipate a participation rate of 80% for our planned survey, leaving us with an *a priori *sample size of 80. The Power Analysis and Sample Size (PASS 2008) software was used to determine the sample size required for testing differences between groups. Assuming a type-one error rate of 5%, a minimal estimated sample size of 51 participants per group will give 80% power to detect a medium effect size using Cohen's guidelines [41]. The sample size for interviews was determined on the basis of Guest's and his colleagues' experiment, which demonstrated that data saturation occurs primarily after 12 interviews (UG) [42], and taking into account multiple investigation sites (PG).

### Analysis

Quantitative data will be managed using the SPSS. We will conduct an assessment of the validity of the questionnaire with our sample using principal component analysis and analysis of internal consistency [43,44]. We will then generate descriptive statistics (frequency, mean, and SD) and perform comparative analyses between groups (using the *t-*test and Mann-Whitney test) to identify PG (T1-T2) differences (p < 0.05, IC 95%) [44]. Qualitative data from interview transcripts and documents will be managed using a formal database using QSR NVivo [45]. Thematic content analysis, using a theoretical orientation strategy, will guide the open-ended coding procedure in order to identify, classify, and reduce data and to build a descriptive matrix [46,47]. The initial coding labels will be established by building on the elements of the OMRU. Table 2 presents a list of these elements and short definitions that describe the coding labels. In order to monitor collaborative practice change following the POBC^3^, we will focus on operational processes (how team members provide care), relational processes (how team members communicate), and adaptive processes (how team members use evidence to enhance collaborative practice) [48].

**Table 2:** Elements of the OMRU with short definitions of coding labels

Elements	Short definitions
**A. Assess barriers and supports**	

Local context	
• Work environment	Factors such as rules, regulations, available resources, and support
• Work pressure	Fit between EIPCP work load and receptivity of involved professionals
• Competing demands	Multiple pressures calling for practice change and importance of EIPCP and time constraints
Recommendations form BPGs	
• Intervention source	Professionals' perception of whether the EIPCP is an externally or internally driven intervention
• Benefits ratio	Professionals' perception of the added value for themselves and for the service users
• Adaptability	The extent to which recommendations can be adapted to fit the dynamics of the local context
• Usefulness	Perceived usefulness of recommendations from BPGs and others sources of evidence
Adopters	
• Knowledge	Professionals' definition and concepts related to collaborative practice and anticipated outcome of EIPCP
• Current practice	Fit between EIPCP, perceived quality of care process and shared decision making
• Beliefs/Attitudes	Value that professionals place on EIPCP and perception of responsibilities regarding care

**B. Monitor degree of use**	

• Operational processes	Sequence of events illustrating how cancer team members perform collaborative care planning and shared decision making,
• Relational processes	Sequence of events illustrating how cancer team members interact, communicate and negotiate shared intervention zone
• Adaptive processes	Sequence of events illustrating how cancer team members enact changes in order to enhance collaborative practices

The trustworthiness of our research data and analysis will be ascertained using Miles and Huberman's criteria of credibility/validity, confirmability/objectivity, and transferability [46]. Given that our study will be an ex-post intervention study [49] and that the sampling method constitutes one of its limitations, the internal validity will be increased by use of a theoretical framework, validated investigation tools previously used by best practice guideline expert researchers, systematic data collection and analysis methods, as well as triangulation [50]. An audit trail of the entire research process will be kept [51].

### Ethics

The study has been approved by the Centre de Recherche de l'Hôpital Charles LeMoyne Ethics Board (ref. number AA-HCLM-09-034).

## Conclusions

This study will constitute a practical trial [52] that takes into account the context-specific challenges and the concerns of nurses and other stakeholders regarding use of evidence-based recommendations. Building on an ongoing improvement initiative, our study represents a unique opportunity for examining the translation of RNAO best practice guidelines into action. POBC^3 ^planned educational workshops and mentoring activities will produce new knowledge on the interventions and sources of support most conducive to the uptake of evidence and building of capacity to sustain new interprofessional collaborative practice patterns. It will provide new information on strategies for overcoming barriers to the adoption of evidence-informed interventions. The findings will also pinpoint new determinants of 'what works and why,' given the interplay between the general application of evidence, uniqueness of cancer patient'/caregivers' needs and preferences, and the local context. It will provide new knowledge on strategies for making care providers aware of evidence-based recommendations from best practice guidelines and others sources of information. This knowledge will contribute to the refinement of continuing education programs, and will add new dimensions to existing survey instruments that assess knowledge, beliefs, and practices regarding evidence-informed interventions.

## Competing interests

The authors declare that they have no competing interests.

## Authors' contributions

The study was initially conceived of by DT and DD. All contributing authors were involved in defining the study design and adapting both the questionnaire and interview grid. The manuscript was written by DT and DD, with all authors both contributing to its development and completion and approving the final version.
